# The Strengths and Difficulties Questionnaire: psychometric properties of the parent and teacher version in children aged 4–7

**DOI:** 10.1186/s40359-015-0061-8

**Published:** 2015-02-20

**Authors:** Lisanne L Stone, Jan M A M Janssens, Ad A Vermulst, Marloes Van Der Maten, Rutger C M E Engels, Roy Otten

**Affiliations:** Behavioural Science Institute, Radboud University Nijmegen, P.O. Box 9104, Nijmegen, 6500 HE The Netherlands; Radboud University Nijmegen, Nijmegen, The Netherlands

**Keywords:** SDQ, Coefficient omega, Construct validity, Measurement invariance, Predictive validity

## Abstract

**Background:**

The Strengths and Difficulties Questionnaire is one of the most employed screening instruments. Although there is a large research body investigating its psychometric properties, reliability and validity are not yet fully tested using modern techniques. Therefore, we investigate reliability, construct validity, measurement invariance, and predictive validity of the parent and teacher version in children aged 4–7. Besides, we intend to replicate previous studies by investigating test-retest reliability and criterion validity.

**Methods:**

In a Dutch community sample 2,238 teachers and 1,513 parents filled out questionnaires regarding problem behaviors and parenting, while 1,831 children reported on sociometric measures at T1. These children were followed-up during three consecutive years. Reliability was examined using Cronbach’s alpha and McDonald’s omega, construct validity was examined by Confirmatory Factor Analysis, and predictive validity was examined by calculating developmental profiles and linking these to measures of inadequate parenting, parenting stress and social preference. Further, mean scores and percentiles were examined in order to establish norms.

**Results:**

Omega was consistently higher than alpha regarding reliability. The original five-factor structure was replicated, and measurement invariance was established on a configural level. Further, higher SDQ scores were associated with future indices of higher inadequate parenting, higher parenting stress and lower social preference. Finally, previous results on test-retest reliability and criterion validity were replicated.

**Conclusions:**

This study is the first to show SDQ scores are predictively valid, attesting to the feasibility of the SDQ as a screening instrument. Future research into predictive validity of the SDQ is warranted.

**Electronic supplementary material:**

The online version of this article (doi:10.1186/s40359-015-0061-8) contains supplementary material, which is available to authorized users.

## Background

In child mental health care and research, screening instruments play an important role in measuring what types of psychosocial problems and strengths may be identified and how severe these problems are, if any. The Strengths and Difficulties Questionnaire (SDQ; [1]) is one of the most widely used screening instruments for these purposes. The SDQ consists of 25 items equally divided across five scales measuring emotional symptoms, conduct problems, hyperactivity-inattention, peer problems, and prosocial behavior. Combining the subscales minus the prosocial scale gives a total difficulties score, indicating the severity and the content of the psychosocial problems. Although much research has been conducted into reliability and validity of the SDQ, several issues warrant further investigation. First, although reliability has been extensively studied see for a review [2], reliability of the subscales seems insufficient, specifically for the conduct problems and peer problems scales. Second, construct validity and measurement invariance have not been examined frequently for both the parent and teacher version, nor for younger children. Third, while stability of SDQ scores over time has been reported [3,4], the degree to which SDQ scores predict subsequent maladjustment has not been examined previously. The goal of the present study was to investigate these three issues. In addition, we present Dutch normative data for the parent and teacher version of the SDQ and report on test-retest reliability and criterion validity.

Regarding reliability, mostly Cronbach’s alphas have been reported (see [2]). Recently, the use of this reliability coefficient has been subject to critique according to psychometricians, due to its underestimation of reliability [5,6], specifically when response scales of items have few categories and when scale distributions are skewed [7]. Evidently, this occurs frequently if not always when measuring psychopathology. Therefore, alternatives to alpha have been suggested and tested, with McDonald’s omega or Jöreskog rho being the most accurate [5]. Employing these accurate measures seems imperative when testing reliability (cf. [8]). Indeed, it has been found that omega coefficients yield higher estimates for the SDQ than alpha [9-12]. Still, these studies are limited by investigating solely the parent version [9,12], relatively small sample sizes [11], and a limited age range, namely preschoolers [10].

Second, support for the SDQ’s five-factor structure is growing as studies increasingly employ confirmatory factor analysis to test its hypothesized factor structure. This is the case for both the parent and teacher version, and for various age ranges [13-18], with only two studies examining this in children aged 4–7 specifically [19,20]. Also, relatively few studies have tested for measurement invariance, namely whether the underlying structure is identical across different groups. Three studies tested measurement invariance for the parent version in older age groups [12,15,16], and two studies in children aged 4–7 [19,21]. These studies found the SDQ to be invariant across gender, age, ethnicity, and maternal education. Regarding the teacher version, two studies tested for measurement invariance in older age groups [16,22], and three studies in children aged 4–7 [19,21,23]. These studies found the SDQ to be invariant across ethnicity, but results are inconsistent regarding gender. Due to the limited number of studies reporting on construct validity and measurement invariance for children aged 4–7 and the inconsistent results on measurement invariance for the teacher version, it was deemed important to investigate these issues in the present study. Measurement invariance is investigated for gender, age, and ethnicity.

Finally, to our knowledge predictive validity has not been investigated for the SDQ. It has been found that SDQ scores predict SDQ scores over a one-year interval [3,4], for both the parent and teacher version. Still, these results do not evidence that SDQ scores are related to a criterion measure over time, they merely show that SDQ scores are correlated over time. Therefore, it was deemed important to investigate the SDQ’s predictive validity in relation to two factors related to child psychopathology; maladaptive parenting and social preference. Specifically, we hypothesized that higher SDQ scores would predict maladaptive parenting and higher parenting stress for the parent version and that higher SDQ scores would predict lower levels of social preference (i.e., the degree to which a child is liked by classmates) for the teacher version.

In the Netherlands, the SDQ is increasingly used to assess psychosocial problems in children. Psychosocial problems in Dutch children are quite common, with the most recent prevalence figures showing that 12% of 5-11-year-olds have psychosocial problems [24]. These problems tend to persist, at least until late childhood [25], and impose a substantial burden on parents [24]. Therefore, it seems important to assess these problems with a well validated instrument with available norms such as the SDQ. However, normative data on Dutch SDQ scores are limited by a small sample size and selectiveness of the sample [26-28]. Therefore, in this paper Dutch normative data are presented for both the parent and teacher version and based on a relatively large sample. In addition, we examined criterion validity in order to replicate previous studies, by comparing SDQ scores to scores obtained by the Child Behavior Check List and Teacher Report Form scores [29]. Similarly, we examined criterion validity for replication purposes for the parent version. Regarding the teacher version, criterion validity has not been extensively investigated [2]. Therefore, we sought to validate the SDQ teacher version by using measures proximal to teachers. Sociometric measures may be particularly useful in this respect, as these may reflect difficulties in peer relations, behaviors exhibited within the school context and are related to child psychopathology (e.g., [30]).

In sum, the present study examined reliability (i.e., Cronbach’s alpha, McDonald’s Omega), test-retest reliability, as well as construct, criterion (concurrent and predictive) validity and measurement invariance of both the parent and teacher version of the SDQ for children aged 4–7. We expected that omega values would yield higher reliability coefficients than alpha. Next, we expected to confirm the hypothesized five-factor structure, to find invariance for gender, age and ethnicity, and we expected substantial inter-correlations among SDQ subscales. Further, we expected that SDQ scores inter-correlate over a retest interval, correlate with similar measures of psychopathology, and are related to maladaptive parenting and sociometric measures within and over time. Finally, we present Dutch normative data for children aged 4–7.

## Methods

### Participants and procedure

Prior to the start of the study ethical approval was obtained from the ethics committee of the Radboud University Nijmegen, reference number ECG05092008. In the 2008–2009 school year, schools were randomly selected from all elementary schools in the Netherlands. Schools in the larger counties (i.e., Noord-Holland, Zuid-Holland, Noord-Brabant, and Gelderland), as well as in the four largest cities (i.e., Amsterdam, Rotterdam, Den Haag, and Utrecht), were oversampled. A total of 440 schools were selected. Directors received a letter in which they were invited to participate in the study. Subsequently, they were called to ask whether they wanted to participate. Directors of 29 schools (6.6%) promised their cooperation. These 29 schools together account for approximately 2300 pupils from the groups 1 to 4. Written informed consent was obtained from the parents of the children who were asked to participate. At the initial measurement, during the 2009–2010 school year, teachers completed the SDQ concerning 2,238 pupils. Regarding the second and third measurement, SDQ data were collected through the teachers about 1,962 and 1,572 pupils, respectively. At the three annual measurement occasions, SDQ data were also collected by means of the parents of the pupils, concerning 1,513, 1,036, and 888 children. Again, at all three annual measurement occasions, sociometric interviews were held with the children themselves, concerning 1,871, 1,603, and 1,770 children. From all these children, 25% came from each of the four groups, and half of the cases concerned boys. Of all children, 79.5% had parents who were both born in the Netherlands, whereas 20.5% had at least one parent who was born abroad (3.5% of Turkish origin, 5.4% Moroccan, and 1.9% Surinam; the remaining children came from parents born in a wide variety of countries). Finally, parents and teachers filled out another SDQ 6 weeks after T1 for 203 and 188 randomly chosen children, respectively, in order to examine test-retest reliability.

## Measures

### Strengths and difficulties questionnaire

The Dutch parent and teacher informant version of the SDQ was used at all waves (SDQ; [31]). The questionnaire consists of five subscales, each of which contain five items measuring emotional symptoms (e.g., many fears, easily scared), conduct problems (e.g., often lies or cheats), hyperactivity-inattention (e.g., restless, overactive, cannot stay still for long), peer problems (e.g., picked on or bullied by other children), and prosocial behavior (e.g., considerate of other people’s feelings). Parents and teachers rated children on a 3-point scale ranging from 0 (not true) to 2 (certainly true). The scoring procedures are available online at http://www.sdqinfo.org.

For each of the five subscales, a score ranges from 0–10 if all five items were completed. Further, a total difficulties score can be calculated by summing the scores from the first four subscales (range 0–40). Mean scores on the SDQ parent version at all measurements in this sample are relatively low for the emotional symptoms scale (range *M*=1.60, *SD*=1.81=*M*=1.67, *SD*=1.87), conduct problems scale (range *M*=1.02, *SD*=1.37–*M*=1.28, *SD*=1.44), hyperactivity scale (range *M*=2.96, *SD*=2.57–*M*=2.98, *SD*=2.50), peer problems scale (range *M*=.98, *SD*=1.39–*M*=1.08, *SD*=1.43), and total difficulties scale (range *M*=6.68, *SD*=5.26–*M*=6.93, *SD*=4.85), and relatively high for the prosocial scale (range *M*=8.16, *SD*=1.72–*M*=8.52, *SD*=1.66). This also holds for the teacher version; emotional symptoms scale (range *M*=1.03, *SD*=1.59–*M*=1.44, *SD*=1.89), conduct problems scale (range *M*=.74, *SD*=1.42–*M*=.82, *SD*=1.31), hyperactivity scale (range *M*=2.64, *SD*=2.83–*M*=2.89, *SD*=2.95), peer problems scale (range *M*=1.05, *SD*=1.51–*M*=1.22, *SD*=1.65), and total difficulties scale (range *M*=5.58, *SD*=4.86–*M*=6.27, *SD*=5.63), and relatively high for the prosocial scale (range *M*=7.67, *SD*=2.35–*M*=8.10, *SD*=2.13). In conclusion, psychosocial difficulties in children between the ages of 4 and 7 are limited in this sample. In fact, we could extend this conclusion to 8 and 9 year-olds, since the oldest children had reached that age at the third measurement.

### Child behavior check list (/1.5-5) and (Caregiver-)teacher report form

The Dutch versions of the CBCL/1.5-5, CBCL, C-TRF and TRF were used to assess internalizing and externalizing behaviour as reported by parents and teachers at T1 [29,32-34]. The CBCL/1.5-5/C-TRF, used for children aged 1.5-5 years, comprises 100 items; the CBCL/TRF targets 5-18-year-olds and consists of 118 items. These items are rated using a 3-point Likert scale, where 0 indicates responses of “not true”, 1 “somewhat or sometimes true”, and 2 “very true or often true”. In all four versions, scores can be calculated regarding internalizing, externalizing and total behavioral problems [35]. The distributions of the scores were skewed, and therefore scores above the 99th percentile were rescaled to the 99th percentile value. Cronbach’s alphas ranged from .84-.87 for the internalizing scale, from .87-.93 for the externalizing scale, and from .91-.94 for the total problems scale, for the parent and teacher version for younger and older children.

### Parenting daily hassles

At all waves parents rated the frequency of daily hassles with their child over the past 6 months (PDH; [35,36]). The questionnaire consists of 20 events of which the parent has to rate how often they occur (seldom, sometimes, often, constantly). A mean score was calculated with higher scores indicating higher parenting stress. Psychometric properties of the PDH have been found adequate [35]. Cronbach’s alphas were .77, .79, and .78 at T1, T2, and T3.

### The parenting scale

The Parenting Scale was used at all waves and asks parents to rate 30 short parenting situations on a 7-point scale (TPS; [37]). Sample items include “When I want my child to stop doing something I firmly tell my child to stop/I coax or beg my child to stop” and “When I’m upset or under stress I am picky and on my child’s back/I am no more picky than usual”. Inadequate parenting behavior is divided across three subscales: permissiveness, restrictiveness, and verbosity. All the items sum up to the total score, which was used in the current study. Higher scores reflect more inadequate parenting behavior. Psychometric properties are adequate [37]. Cronbach’s alphas were .77, .81, and .80, for the total score at T1, T2 and T3.

### Social preference

At all waves children were interviewed individually. During these interviews, children were shown a photograph of their classmates. A trained research assistant pointed out a child on the photograph and asked the child whether (s) he knew who this child was, ensuring familiarity, and was then asked whether (s) he liked, disliked the child or thought neutral of him/her. To increase comprehension and ease shy children, the child could respond verbally or by pointing to three fluffy smileys, with either a happy, sad or neutral expression. This procedure was repeated until the child gave a nomination bout every child in the class. The order of asking questions about children in the photograph was counterbalanced, such that the interviewer started either at the upper left, upper right, lower left or lower right corner of the photograph. Unlimited nominations (like, dislike, neutral) were used, because these tend to spread more evenly among children in a class than limited nominations (i.e., fewer children receive a raw nomination score of zero). For each child, scores were calculated that indicate the extent to which a child is liked by fellow pupils (‘Like-score’), and the extent to which fellow pupils do not like the child (‘Dislike-score’). These scores were standardized within each classroom. The total least-liked nomination was subtracted from the total most-liked nomination to obtain a measure of social preference (cf. [38]). These scores were obtained at T1, T2, and T3.

### Strategy for analysis

For the SDQ, we computed the reliability measure of Cronbach’s alpha. Also, we computed rho of Jöreskog [39], also known as McDonald’s omega [40,41]. This measure shows the relationship between the variance explained by a factor and the total amount of variance to be explained by that factor, and has been recommended to be used [8]. Research in which omega is applied to the SDQ, has shown good results [12,42]. Reliability measures less than 0.70 are considered moderate, reliability measures between 0.70 and 0.80 are regarded sufficient, and measures above 0.80 are good [43]. Furthermore, we computed Spearman’s rho correlations between SDQ scales at T1 and SDQ scales completed after a retest interval of 6 weeks in order to examine test-retest reliability. In all analyses a two-tailed significance level was used.

Construct validity was examined using confirmatory factor analysis (CFA). By means of CFA, it was tested whether the assumed five factor model of the SDQ could be confirmed, using Mplus [44]. For brevity reasons, for a detailed description of our analytical strategy regarding CFA we refer to [12]. Model fit was assessed with various fit indices, including robust chi-square with estimated degrees of freedom (df), comparative fit index (CFI; [45]), and root mean squared error of approximation (RMSEA; [46]). It is assumed that a factor model has a good fit when CFI > .95 en RMSEA < .05 and is acceptable when CFI > .90 en RMSEA < .08 [47].

Criterion validity is present when the score corresponding to an instrument is related to the score on an external criterion (an existing valid instrument) that measures the same property. The SDQ is valid when scores on the SDQ correlate sufficiently high with scores produced by other instruments that also measure psychosocial problems in children. Correlations < .30 are considered low, ≥ .30 average/medium, and ≥ .50 high [48].

To investigate the predictive validity of the SDQ, we used Growth Mixture Modeling (GMM) [44]. By means of GMM, developmental profiles can be established, based on the SDQ scores at the three points in time. By doing so, we considered the development of the SDQ scores over time, instead of studying a single score at one moment in time. These profiles are constructed on the basis of growth parameters of the SDQ scores over the three measurements. In this case, these growth parameters consist of the intercept and the slope^a^. The intercept can be regarded as the initial level of the SDQ scores. The slope represents the degree of change of these scores over time. To investigate the number of different profiles that are present in the population to be studied, we examined the most obvious ‘solution’, according to the fit statistics and theory. Several fit statistics are available, on the basis of which the best fitting number of profiles can be determined: The BIC (Bayesian Information Criterion), and the AIC (Akaike Information Criterion) [49]. The model presenting the lowest value shows the best fit. The entropy value shows a good fit when being equal to or above 0.80. Subsequent to the identification of developmental profiles, one-way univariate ANOVA’s were conducted to test whether these groups differed on parenting measures and social preference scores. A Bonferroni correction was used to correct for multiple testing.

## Results

### Reliability

The results with respect to reliability are presented in Table 1. Cronbach’s alpha ranges from .46 to .82 for the parent version, and from .53 to .88 for the teacher version. McDonald’s omega ranges from .67 to .90 for the parent version, and from .82 to .93 for the teacher version. We may conclude that the reliability indexed by Cronbach’s alpha is insufficient for the conduct problems, peer problems, emotional symptoms and prosocial scales of the SDQ parent version, while reliability indexed by McDonald’s omega yields sufficient to good estimates for all subscales. Reliability indexed by Cronbach’s alpha of the teacher version is insufficient for the conduct problems and peer problems scales, and good for all subscales when indexed by McDonald’s omega.

**Table 1:** **Cronbach’s Alpha and McDonald’s Omega for the SDQ subscales for the parent and teacher version**

**SDQ parent**	**Measurement 1**		**Measurement 2**		**Measurement 3**	
	**α**	**ω**	**α**	**ω**	**α**	**ω**
Emotional symptoms	.63	.79	.67	.82	.66	.81
Conduct problems	.48	.70	.46	.67	.55	.77
Hyperactivity	.77	.86	.79	.88	.81	.89
Peer problems	.51	.73	.54	.75	.63	.81
Prosocial behavior	.61	.75	.67	.81	.68	.82
Total difficulties	.77	.87	.78	.89	.82	.90
**SDQ teacher**	**Measurement 1**		**Measurement 2**		**Measurement 3**	
	**α**	**ω**	**α**	**ω**	**α**	**ω**
Emotional symptoms	.71	.87	.75	.89	.72	.87
Conduct problems	.53	.85	.68	.85	.73	.89
Hyperactivity	.83	.92	.88	.95	.88	.95
Peer problems	.64	.82	.67	.82	.67	.82
Prosocial behavior	.81	.89	.82	.90	.81	.90
Total problems	.80	.91	.85	.93	.85	.93

Furthermore, test-retest reliability of the parent version was examined, with correlations of .77 for the total problems scale, .81 for hyperactivity-inattention, .72 for emotional problems, .72 for prosocial behaviour, .54 for peer problems and .55 for conduct problems. For the teacher version, correlations of .80 for the hyperactivity-inattention and total problems scales, .77 for emotional problems, .70 for prosocial behaviour, .65 for peer problems and .58 for conduct problems were found. All correlations were significant at *p* < .001.

### Construct validity

It was examined whether the meaning of the five SDQ subscales is equivalent across several important characteristics (i.e., gender, age, and ethnicity), which is referred to as measurement invariance. It is not intended that the meaning of, for example, Emotional symptoms, is different for the 4–5 year olds than for the 6–7 year olds. The procedure applied and the corresponding outcomes are specified in Additional file 1. Based on the outcomes, we may conclude that the construct validity is not different regarding gender, age, and ethnicity, for the parent version of the SDQ. The comparison between boys and girls, older and younger children, and native and non-native Dutch is thus justified. Concerning the teacher version, the most stringent form of measurement invariance was not established for gender, while this was established for age and ethnicity.

Because support was found for the first type of measurement invariance, configural invariance, a final CFA was conducted over all participants. The fit of the final CFA model with regard to the parent version was *χ*2(265)=1314.60, *p*=0.000, CFI=.885, RMSEA=.051 at first measurement, *χ*2(265)=945.43, *p*=0.000, CFI=.900, RMSEA=.050 at second measurement, and *χ*2(265)=821.59, *p*=0.000, CFI=.924, RMSEA=.048 at third measurement, indicating that the parent version of the SDQ thus has an acceptable fit. This means that the five theoretically supposed scales are empirically demonstrable. The fact that the fit is good at three different measurements, further shows that there is robustness of the factor structure. After all, this is demonstrated at different points in time. Results of the factor analyses regarding the SDQ parent version, are presented in Table 2 in terms of standardized loadings. The factor loadings are adequate, that is to say, larger than or equal to .40, although a few loadings are somewhat smaller. These are the items ‘Often complains of headaches, stomach-aches, or nausea’ (somatic) from the Emotional symptoms scale, and ‘Steals from home, school or elsewhere’ (steals) from the Conduct problems scale.

**Table 2:** **Factor loadings of the parent and teacher version of the SDQ**

	**Factor loadings**					
	**Parent**			**Teacher**		
	**T1**	**T2**	**T3**	**T1**	**T2**	**T3**
*Emotional symptoms*						
Somatic	.39	.46	.36	.53	.54	.56
Worry	.72	.77	.75	.73	.77	.76
Unhappy	.84	.78	.82	.90	.94	.92
Clingy	.66	.72	.71	.81	.82	.74
Fears	.63	.67	.73	.78	.80	.75
*Conduct problems*						
Tantrums	.59	.64	.67	.71	.67	.79
Obedient*	.61	.60	.68	.64	.87	.84
Fights	.73	.61	.65	.80	.82	.83
Lies	.52	.55	.66	.76	.77	.81
Steals	.35	.28	.49	.72	.51	.65
*Hyperactivity*						
Restless	.77	.82	.79	.91	.93	.92
Fidgeting/squirming	.73	.73	.76	.85	.90	.91
Distracted	.80	.82	.90	.85	.90	.88
Thinks*	.62	.67	.67	.77	.83	.85
Completes*	.76	.79	.81	.76	.87	.85
*Peer problems*						
Solitary	.51	.45	.56	.52	.46	.45
Good friend*	.44	.57	.59	.71	.76	.70
Popular*	.81	.84	.85	.97	.99	.98
Bullied	.61	.57	.70	.66	.67	.69
Good with adults	.59	.63	.66	.54	.50	.55
*Prosocial behavior*						
Considerate	.79	.89	.90	.93	.97	.98
Shares	.59	.68	.64	.79	.81	.80
Helpful	.59	.62	.64	.80	.83	.79
Kind	.52	.61	.67	.71	.71	.76
Helps	.57	.55	.61	.72	.71	.63

*Note.* Items marked with an asterisk are reversed items.

The fit of the CFA with regard to the teacher version was *χ*2(265)=2619.55, *p*=0.000, CFI=.920, RMSEA=.063 at the first measurement, *χ*2(265)=2930.75, *p*=0.000, CFI=.930, RMSEA=.071 at the second measurement, and *χ*2(265)=2330.38, *p*=0.000, CFI=.930, RMSEA=.070 at the third measurement. Like the parent version, the teacher version of the SDQ has an acceptable fit. This means that the five theoretically supposed scales are empirically demonstrable in this case as well. Again, robustness of the factor structure is demonstrated by showing that the structure is identified at three time-points. Standardized loadings are reported in Table 2. A table wherein the mutual correlations between the latent five factors with regard to the parent and teacher version of the SDQ are displayed is available upon request from the first author.

### Criterion validity: correlations between SDQ and CBCL/TRF

The scores on the CBCL/TRF scales are correlated with the scores on the SDQ scales. SDQ Total Difficulties scores correlate strongly with the CBCL and TRF total problems scores. The SDQ subscale Emotional symptoms correlates highly with the Internalizing problems scale as measured by the CBCL and TRF. The SDQ scales that point to externalizing problem behavior (Conduct problems, Peer problems, and Hyperactivity) are closely related to the CBCL and TRF Externalizing problems scale. All of these high correlations indicate a high degree of SDQ criterion validity. The table wherein the results are presented is available upon request from the first author.

### Criterion validity: correlations among SDQ subscales and SDQ scales with parenting measures

First, we examined whether the subscales of the parent and teacher version are correlated. We found low but significant (*p* < .01) correlations for Emotional symptoms (.26), Conduct problems (.29), and Prosocial behavior (.21), and medium for Peer problems (.32), Hyperactivity (.48) and Total difficulties (.40).

Second, we examined whether SDQ scores were related to scores associated with psychosocial problems. It was expected that as parents raise their children more inadequate, these children would score higher on the SDQ problem scales. Obviously, this hypothesis especially concerned the parent version of the SDQ, yet we also checked whether high scores on inadequate parenting behavior were related to high scores on the SDQ problem scales of the teacher version. If we would find these correlations, than that too would be indicative of the criterion validity of the SDQ teacher version. In Table 3, correlations between the SDQ scores and scores on the TPS and PDH are presented. All subscales of the SDQ parent version are significantly correlated with the TPS scores (range .13-.24) and with the PDH scores (range .22-.40). Highest correlations were found between Total difficulties and the TPS- and the PDH-score, respectively .24 and .40. It appears that the less adequate parents raise their children, the more problems these children exhibit, and that the more problems children exhibit, the greater parents’ daily hassles tend to be. The SDQ scales of the teacher version are hardly associated with the TPS scores. However, these scales are associated with PDH scores. The correlations are low, albeit in the expected direction. As children are experienced by their teachers as more problematic, parents of these children experience more daily hassles.

**Table 3:** **Correlations between SDQ scores and scores on The Parenting Scale (TPS), Parenting Daily Hassles (PDH) and sociometric measures**

**Parent**	**TPS**	**PDH**	**Like**	**Dislike**	**Social preference**
Emotional symptoms	0.13**	0.23**	−0.07*	0.04	−0.06*
Conduct problems	0.23**	0.35**	−0.22**	0.23**	−0.24**
Hyperactivity	0.18**	0.29**	−0.26**	0.24**	−0.26**
Peer problems	0.16**	0.22**	−0.23**	0.20**	−0.23**
Prosocial behavior	−0.14**	−0.23**	0.15**	−0.13**	0.15**
Total difficulties	0.24**	0.40**	−0.29**	0.26**	−0.29**
**Teacher**	**TPS**	**PDH**	**Like**	**Dislike**	**Social preference**
Emotional symptoms	0.02	0.06*	−0.09**	0.07**	−0.08**
Conduct problems	0.06*	0.11**	−0.33**	0.37**	−0.37**
Hyperactivity	0.04	0.09*	−0.35**	0.36**	−0.38**
Peer problems	0.02	0.14**	−0.33**	0.31**	−0.34**
Prosocial behavior	−0.04	−0.15**	0.32**	−0.30**	0.33**
Total difficulties	0.04	0.10*	−0.41**	0.42**	−0.43**

*p < 0.05, **p < 0.01.

Finally, the SDQ’s criterion validity was examined by relating SDQ scores to like, dislike and social preference scores. These three scores correlate–0.41, 0.42, and–0.43, respectively, with the SDQ Total Difficulties score of the teacher version, and–0.29, 0.26, and–0.29 with the Total Difficulties score of the parent version. Equivalent correlations apply to the SDQ subscales (see Table 3). Hence, it appears that as pupils exhibit more psychosocial problems, they are less liked by their classmates. In conclusion, we may state that–with the findings above–the criterion validity of the SDQ is amply demonstrated.

### Criterion validity: predictive validity

Finally, the predictive validity was studied by examining whether developments in the course of SDQ scores over three measurements, were predictive for the course of inadequate parenting behavior and daily parenting hassles for the parent version, and were predictive of social preference scores for the teacher version, over the same period of time. Predictive validity is present when SDQ scores are predictive of scores on these parenting and social preference measures.

**Figure 1:**
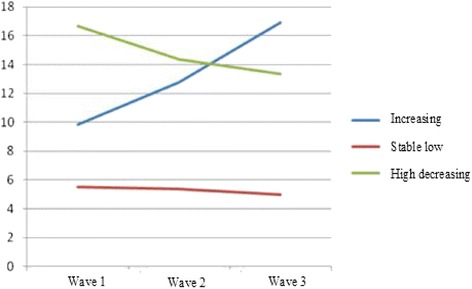
**Developmental profiles SDQ (parent version).**

At the first step, we tested which model fitted the data best, using GMM. As can be seen in Table 4, when taking all fit statistics in consideration (i.e., relatively low levels of the AIC and BIC combined with a good entropy), these call for a model providing three developmental pathways. One large group scores consistently low on the SDQ total score (85.7%); one group scores high and demonstrates a slight decrease over time (5.1%); and one group that starts somewhat lower than the previous group, but shows a small increase over time (9.1%). These pathways are illustrated in Figure 1.

**Table 4:** **Fit statistics for developmental profiles for the parent and teacher version of the SDQ**

	**Parent version**			**Teacher version**		
	**AIC**	**BIC**	**Entropy**	**AIC**	**BIC**	**Entropy**
1 profile	19499	19542	1.00	34480	34527	1.00
2 profiles	19242	19301	0.84	34008	34072	0.84
3 profiles	19166	19241	0.80	33806	33888	0.82
4 profiles	19083	19174	0.76	33699	33798	0.80
5 profiles	19063	19108	0.77	33630	33746	0.77
6 profiles	19069	19194	0.67	33552	33686	0.72

At the second step, the developmental pathways were linked to scores on TPS and PDH. Results are presented in Table 5. The findings show that developmental pathways of the SDQ are associated with scores on TPS, with significantly higher scores in the high-decreasing group as compared to the stable-low group. At time three there was an overall significant effect (*p*=.045). However post-hoc tests (Bonferroni) revealed no significant differences between the different groups. Regarding daily hassles, at the time of the first measurement, the three groups all differed significantly from each other. In the second and third measurement only the stable-low group and the high-decreasing group differed significantly. Strikingly, the two high trajectories hardly differ from each other with regard to the parenting measures, differences mainly exist between the large group exhibiting few problems and the two high trajectories. Less inadequate parenting behavior occurs and less daily hassles are experienced in the group exhibiting few problems, as compared to the other two groups. In sum, we can conclude that the SDQ demonstrates predictive validity in a sense that higher levels of psychopathology over time are generally associated with more parenting problems and daily hassles.

**Table 5:** **Relationships between the course of SDQ scores (parent and teacher version) and the course of parenting and social preference**

	**Parent version**			
	**Stable-low**	**Increasing**	**High-decreasing**	
Parenting measures	*M (SD)*	*M (SD)*	*M (SD)*	
Parenting scale T1	2.90 (0.45) a	2.98 (0.49)	3.10 (0.47) a	*F*=12.025, *p*=0.000
Parenting scale T2	2.86 (0.45) a	2.96 (0.49)	3.04 (0.54) a	*F*=6.520, *p*=0.002
Parenting scale T3	2.84 (0.45)	2.96 (0.49)	2.94 (0.47)	*F*=3.123, *p*=0.045
Daily Hassles T1	1.46 (0.24) ab	1.61 (0.28) ac	1.72 (0.32) bc	*F*=73.274, *p*=0.000
Daily Hassles T2	1.46 (0.24) ab	1.70 (0.32) a	1.73 (0.29) b	*F*=63.997, *p*=0.000
Daily Hassles T3	1.42 (0.22) ab	1.67 (0.31) a	1.65 (0.28) b	*F*=55.179, *p*=0.000
	**Teacher version**			
Social Preference	*M (SD)*	*M (SD)*	*M (SD)*	
Social Preference T1	0.14 (0.91) ab	−0.65 (0.95) b	−0.87 (0.98) a	*F*=118.55, *p*=0.000
Social Preference T2	0.16 (0.89) ab	−0.70 (1.02)	−0.60 (1.00) a	*F*=71.72, *p*=0.000
Social Preference T3	0.12 (0.92) ab	−0.82 (0.91) b	−0.61 (1.06) a	*F*=79.16, *p*=0.045

*Note* The lowercase letters a and b indicate which groups differ on the relevant variable. For example, parenting at measurement 1: The Stable-low group differs from the High-decreasing group, but not from the Increasing group, nor does the Increasing group differ from the High-decreasing group.

**Figure 2:**
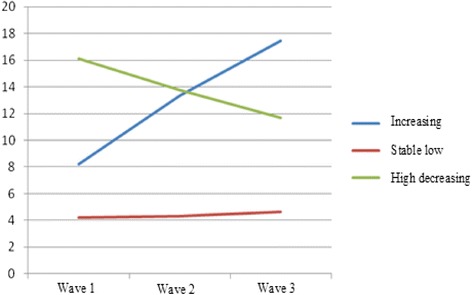
**Developmental profiles SDQ (teacher version).**

In order to further investigate the predictive validity of the SDQ teacher version, the degree of coherence between the developmental pathways of SDQ scores and the scores that are indicative of the children’s likability, namely social preference, was examined. Again, we used GMM at the first step to test which model fitted the data best. Table 6 shows that when all fit statistics are taken into consideration these again argue for a model providing three developmental pathways. This can also be seen in Figure 2: One large group scores consistently low on the SDQ total score (81.4%); one group scores high and demonstrates a slight decrease over time (8.7%); and one group that starts somewhat lower than the previous group, but shows a small increase over time (9.9%).

**Table 6:** **Dutch normative data for the parent and teacher version of the SDQ: Subclinical and clinical scores for children aged 4-7**

		**Parent**			**Teacher**		
		**Total**	**Boys**	**Girls**	**Total**	**Boys**	**Girls**
*Emotional symptoms*	Subclinical	4	4	4	3	3	3
	Clinical	5-10	5-10	5-10	4-10	4-10	4-10
*Conduct problems*	Subclinical	3	3	3	2	3	2
	Clinical	4-10	4-10	4-10	3-10	4-10	3-10
*Hyperactivity*	Subclinical	6	7	6	7	8	5-6
	Clinical	7-10	8-10	7-10	8-10	9-10	7-10
*Peer problems*	Subclinical	3	3	3	3	3-4	3
	Clinical	4-10	4-10	4-10	4-10	5-10	4-10
*Prosocial behavior*	Subclinical	5	4	5	3	3	4
	Clinical	0-4	0-3	0-4	0-2	0-2	0-3
*Total difficulties*	Subclinical	14-16	14-16	13-15	12-14	14-16	12-13
	Clinical	17-40	17-40	16-40	15-40	17-40	14-40

At the second step, the developmental pathways were linked to the social preference scores. The results are presented in Table 5. These clearly show that developmental pathways of the SDQ as indicated by teachers, are associated with the extent to which children are liked by their classmates. Hence, the SDQ teacher version demonstrates predictive validity as well. Again, it is noticeable that the two high trajectories hardly differ with regard to social preference scores. The children in the large, stable group are most liked by their classmates.

### Normative data

Finally, normative data are presented for the Dutch population, and for both the parent and teacher version of the SDQ for children aged 4–7. For each child from our sample, we calculated the score on every SDQ subscale and the Total Difficulties score at T1. For each of the five scales, scores vary between 0–10; for Total Difficulties between 0–40. A cumulative percentage equal to or over 95% corresponding to a certain score, means that in the normative sample, 95% of the children acquired lower scores than the child who obtained that particular score, or stated differently, the child belongs to the 5% children exhibiting most problems on that scale. This is referred to as a clinical score. A score corresponding to a cumulative percentage between 90 and 95% is called a subclinical score. Such a score implies that a child belongs to the 10% children exhibiting most problems. Next, we repeated this procedure separately for boys and girls. In Table 6, the scores of the total sample and the scores specified by gender are presented. To facilitate interpretation, we summarized when scores are considered subclinical and clinical as to the five subscales and Total Difficulties score. Generally, it can be stated that the normative scores for the subgroups based on gender, hardly differ from those for the total sample.

## Discussion

In the present study, psychometric properties of the parent and teacher version of the SDQ were examined for children aged 4–7 in a large sample. Specifically, omega coefficients and most test-retest indices were adequate, the five-factor structure was confirmed, and indices of criterion validity were adequate. Next, support for measurement invariance was strongest for gender and age, and less so for ethnicity for the parent version. Regarding the teacher version, support for the most stringent type of measurement invariance was not strong across time points, although the less stringent type of measurement invariance was established for age, gender and ethnicity. Further, our results supported the predictive validity of the SDQ. Finally, normative data for the Dutch population were presented. Generally, the SDQ’s psychometric properties can be classified as adequate in this community sample, in young children and with the goal of the SDQ as a screening instrument. Specifically, psychometric properties of the SDQ are dependent on characteristics of the sample and the goal of this study [50]. With these notions being made, this study is the first to examine predictive validity of the SDQ while also comprehensively assessing several modern indicators of reliability and validity. As such, this study is an important contribution to the psychometric literature on the SDQ.

In line with our expectations regarding reliability, we found consistently higher omega coefficients than Cronbach’s alpha coefficients. These results mesh with previous studies investigating omega and alpha [12,42]. With the relatively low alpha coefficients being reported previously it has been argued to refrain from using the separate subscales of the SDQ, specifically so for the conduct problems and peer problems scales [2,19]. However, we showed that these subscales seem to be reliable when an indicator of reliability is employed that takes skewness and difficulties due to limited response categories into account. Therefore, we argue that scores from separate subscales are reliable and thus can be interpreted.

Second, as expected, we were able to confirm the five-factor structure of the SDQ for both the parent and teacher version, which is in line with previous studies employing CFA [13-20]. Also, we found SDQ scores to be at least configurally invariant across gender, age, and ethnicity for the parent version of the SDQ. On a scalar and metric level SDQ scores were also invariant for gender. These results are largely in line with previous studies [12,15,16,19,21]. For the teacher version, SDQ scores were also configurally invariant across age, gender and ethnicity. However, for gender, invariance was not established on a scalar and metric level. These inconsistent results are in line with previous studies on the teacher version [16,19,21-23]. Also, support for scalar and metric invariance was somewhat inconsistent across time points for both the parent and teacher version regarding age and ethnicity. Further research is warranted on measurement invariance regarding both the parent and teacher version in order to further clarify these inconsistent results.

Regarding predictive validity, we showed inclusion in a risk-group (i.e., the highest SDQ scores in the sample) was predictive of more maladaptive parenting and higher degrees of parenting stress. Also, we found inclusion in a risk-group predictive of lower degrees of being liked, in other words, children who were rated as having more psychosocial problems were less liked by their peers. These results are particularly important for the viability of the SDQ as a screening instrument, as they show that SDQ scores are related to other types of maladjustment over time, attesting to the robustness of the SDQ.

As for criterion validity, we showed that SDQ scores for the parent version were consistently related to maladaptive parenting and parenting stress. Scores on the teacher version were not strongly related to the parenting measures, but were to the sociometric measures. Specifically, the sociometric measures, being liked, disliked and social preference (i.e., the degree to which the child is liked by peers) correlated substantially with parent and teacher rated scores. These results confirm the criterion validity of SDQ scores for both the parent and teacher version. Moreover, given the stability typically found in sociometric measures [51], these measures may be very suited as criterion measures for validation purposes in future studies.

Finally, we presented normative data for children aged 4–7 for the Dutch version of the SDQ enabling researchers and clinicians to interpret SDQ scores as being ‘normal’, ‘subclinical’ or ‘clinical’. When comparing these results to British, Danish and Swedish normative data, our results are largely in line with these studies for both the parent and teacher version [52-54]. With the presentation of these norms we facilitate the use of the SDQ as a screening instrument in young children where the potential of prevention and intervention are high. Particularly in these young children this potential may be high as problems have probably not yet fully become integrated into the child’s personality. Still, our results show that a small group of children increases in their problem levels. Therefore, targeting such an at-risk group in particular seems a fruitful approach for prevention and intervention.

Some limitations of this study should be noted. First, we did not investigate psychometric properties of the SDQ in a clinical sample and therefore do not know whether our results may be generalized to such a population. As the SDQ is used frequently in clinical practice, either as part of screening at intake or as a routine outcome monitoring instrument (e.g., [55]), this is an important avenue for future research. Also, although we specifically focused on young children due to limited research concerning this age group, the normative data presented may be quite limited, especially to clinicians. If normative data were established for the complete age range of the SDQ, this would be very useful to clinicians. Second, in this study relatively high attrition levels were found, possibly compromising our results regarding predictive validity and measurement invariance. Therefore, future research into predictive validity and measurement invariance is warranted to replicate our findings. Clinical diagnoses or alternative measures of child adjustment could be included in future studies to examine whether SDQ scores predict maladjustment on these measures.

## Conclusions

Despite the aforementioned limitations, this study adds to the literature by showing that key aspects of psychometrics, namely reliability, construct validity, measurement invariance and predictive validity were found adequate for the parent and teacher version of the SDQ in this community sample.

## Endnotes

^a^As for the findings described here, a study consisting of three measurements was used. Therefore, only linear development of problem behavior could be examined. When having data on multiple measurements, one can take a look at development using quadratic or cubic models, for example.

## Additional file

Additional file 1:
**Measurement invariance analysis [** 56 **-** 62 **].**
