# Supplementary material for: The Strengths and Difficulties Questionnaire: psychometric properties of the parent and teacher version in children aged 4–7
Source: BMC Psychol. 2015 Feb 20;3(1):4. doi: 10.1186/s40359-015-0061-8 (PMC4364334; doi:10.1186/s40359-015-0061-8)
Supplement: Additional file 1: — Measurement invariance analysis [ 56 - 62 ]. [file 40359_2015_61_MOESM1_ESM.doc]

**Appendix 1 Measurement invariance analysis**

Measurement invariance means that the construct validity is the same for different groups. This includes, for example, the question whether the meaning of the Emotional symptoms scale is the same for boys and girls. In this study, it is assessed whether measurement invariance can be determined with regard to gender, age, and ethnicity. When the measurement invariance of an instrument is established, this allows for meaningful group comparisons [e.g., 56, 57]. Three forms of measurement invariance are distinguished: configural invariance, metric invariance, and scalar invariance [58]. *Configural invariance* refers to the question of whether the factors comprise the same set of items for each of the subgroups. Configural invariance is supported if the baseline model fit is acceptable or good. The baseline model is a combination of factor analyses regarding every subgroup: Each subgroup yields a certain fit, that is combined in an aggregated fit across all subgroups. To meet the requirement of configural invariance, this aggregated fit needs to be acceptable or good. *Metric invariance* means that it is tested whether the loadings are the same across groups. In principal, this is done by equating the loadings across groups, and comparing the fit of this model to the fit of the baseline model. If the fit does not significantly worsen, this supports metric measurement invariance. A more stringent form of measurement invariance concerns the item intercepts. If we consider that the factor model resembles a regression model in which item scores are estimated by the corresponding latent factor, then such a regression model contains intercepts and regression weights. The regression weights are the loadings that, in the previous step, were equated. On top of that, the intercepts are now equated. This is called *scalar invariance.* The fit of this scalar invariance model is compared to the fit of the previous (metric invariance) model, and it is expected that the fit will not significantly worsen.

For evaluating measurement invariance of scales based on ordinal items (as is the case here), the matter is more complex. Instead of intercepts and factor loadings, we now have to deal with thresholds and factor loadings. Metric and scalar invariance can now only be examined simultaneously [44, 59]. For this purpose, the configural invariance model is compared to the model in which factor loadings and thresholds are equated simultaneously for different groups. To test whether these restriction worsened the model, a chi-square difference test was applied, as indicated on the Mplus website (<http://www.statmodel.com/chidiff.html>). However, when the chi-square difference test is applied to nested models, as in this case, the test is directly influenced by the sample size. Trivial differences can still be significant to large sample sizes [60], and this test is therefore seen as highly sensitive, but not very practical [61]. For that reason, next to this chi-square difference test, we also viewed the fit indices, as is recommended [61]. When the chi-square difference test value increases no more than .01 from the baseline model to the restricted model, it can be argued that measurement invariance should not be rejected [cf. 12].

The items of the SDQ have 3-points response scales and are mostly very positively skewed. For this type of items (denoted as ordered categorical in Mplus) we used the Weighted Least Square estimator with Mean- and Variance-adjusted chi-square test statistic (WLSMV). Scholars have investigated the consistency of the WLS(MV)-estimator under various missing data assumptions and concluded that using all available pairwise information in the data produced unbiased and efficient estimates for the parameters to be estimated [62].

**Parent version**

The five factor model was tested, and factor loadings and thresholds were left free to vary. Table 1 shows that the configural invariance of the SDQ parent version is supported at the three measurements with respect to gender, age, and ethnicity (Model 1). The fit is adequate; CFI > .887 and RMSEA < .052. Factor loadings and thresholds were equated to test metric and scalar invariance (Model 2). In Table 1, the fit indices are presented.

**T1.** Regarding gender, the chi-square difference test results showed no significant difference between Model 1 and Model 2 (Δχ²(70) = 86.11, p = .09). The DIFF test further indicated significant differences regarding age (Δχ²(215) = 338.04, p = .000) and ethnicity (Δχ²(70) = 172.45, p = .000). However, when viewing the differences in the CFI values, it can be seen that these increase no more than .01. Therefore, it can be concluded that there are no substantial differences between the models. Measurement invariance is thus supported as regards gender, age, and ethnicity.

**T2.** Regarding gender, the DIFF test turned out insignificant (Δχ²(70) = 89.52, p = .058), meaning that Model 1 and 2 do not differ from one another and invariance is supported. It appeared that at T2, there were not enough observations on certain items with respect to age and ethnicity, impeding model identification. Measurement invariance is thus not established regarding age and ethnicity at this time point.

**T3.** The DIFF test proved significant regarding gender (Δχ²(70) = 108.26, p = .002), and age (Δχ²(215) = 302.01, p = .000). However, the increase in CFI values was no more than .01. It can therefore be concluded that the models do not substantially differ from each other, and measurement invariance is thus supported with regard to these variable. It turned out that at T3, there were not enough observations on certain items as regards ethnicity, hindering model identification. Measurement invariance is thus not established regarding ethnicity at T3.

**Teacher version**

The five factor model was tested, and factor loadings and thresholds were left free to vary. Table 2 shows that configural invariance of the SDQ teacher version is supported at the three measurements with respect to gender, age, and ethnicity (Model 1). The fit is acceptable; CFI > .900 and RMSEA < .070. Factor loadings and thresholds were equated to test metric and scalar invariance (Model 2). In Table 1, the fit indices are presented.

**T1.** Regarding ethnicity, the DIFF test proved significant (Δχ²(70) = 121.50, p = .000), but the increase in CFI was no more than .01. Therefore, it can be concluded that Model 1 and 2 do not differ from one another, and measurement invariance is thus supported as regards ethnicity. With respect to gender, the DIFF test turned out significant as well (Δχ²(70) = 124.60, p = .000), and the increase in CFI is somewhat larger than .01. Metric and scalar invariance are thus not completely supported as regards gender. It appeared that at T1, there were not enough observations on certain items with respect to age, impeding model identification. Measurement invariance is thus not established regarding age at this time point.

**T2.** The DIFF test proved significant regarding gender (Δχ²(70) = 138.81, p = .000), age (Δχ²(215) = 346.86, p = .000), and ethnicity (Δχ²(70) = 116.76, p = .000). The increase in CFI was larger than .01. Metric and scalar invariance are thus not supported as regards these variables.

**T3.** Regarding age and ethnicity, the DIFF test turned out significant (Δχ²(215) = 442.02, p = .000; and Δχ²(70) = 129.86, p = .000, respectively). The increase in CFI, however, appeared to be no more than .01. Therefore, it can be concluded that there are no substantial differences between Model 1 and 2. Measurement invariance is thus supported as regards age and ethnicity. The DIFF test proved significant for gender as well (Δχ²(70) = 125.72, p = .000), yet the increase in CFI was somewhat larger than .01. Therefore, metric and scalar invariance are not completely supported with respect to gender.

Table 1 Fit indices of the SDQ parent version

| Model | Factor loadings  and thresholds | Variable | χ^2^ | df | p | CFI | RMSEA |
| --- | --- | --- | --- | --- | --- | --- | --- |
| 1 | Free to vary | Gender | 1467.50 | 530 | .000 | .879 | .050 |
| 2 | Equal |  | 1430.83 | 600 | .000 | .893 | .044 |
| 1 | Free to vary | Age | 1976.73 | 1060 | .000 | .880 | .049 |
| 2 | Equal |  | 2156.41 | 1275 | .000 | .884 | .044 |
| 1 | Free to vary | Ethnicity | 1314.63 | 530 | .000 | .882 | .049 |
| 2 | Equal |  | 1367.27 | 600 | .000 | .885 | .045 |
| T2 |  |  |  |  |  |  |  |
| 1 | Free to vary | Gender | 1116.44 | 530 | .000 | .897 | .047 |
| 2 | Equal |  | 1121.78 | 600 | .000 | .908 | .042 |
| T3 |  |  |  |  |  |  |  |
| 1 | Free to vary | Gender | 999.62 | 530 | .000 | .927 | .045 |
| 2 | Equal |  | 1035.68 | 600 | .000 | .932 | .041 |
| 1 | Free to vary | Age | 1665.64 | 1060 | .000 | .908 | .051 |
| 2 | Equal |  | 1837.43 | 1275 | .000 | .915 | .045 |

Table 2 Fit indices of the SDQ teacher version

| Model | Factor loadings  and thresholds | Variable | χ^2^ | df | p | CFI | RMSEA |
| --- | --- | --- | --- | --- | --- | --- | --- |
| 1 | Free to vary | Gender | 2793.89 | 530 | .000 | .917 | .062 |
| 2 | Equal |  | 2550.15 | 600 | .000 | .928 | .054 |
| 1 | Free to vary | Ethnicity | 1945.32 | 530 | .000 | .932 | .055 |
| 2 | Equal |  | 1832.14 | 600 | .000 | .941 | .048 |
| T2 |  |  |  |  |  |  |  |
| 1 | Free to vary | Gender | 2692.35 | 530 | .000 | .928 | .068 |
| 2 | Equal |  | 2409.60 | 600 | .000 | .940 | .058 |
| 1 | Free to vary | Age | 3520.41 | 1060 | .000 | .928 | .072 |
| 2 | Equal |  | 3285.75 | 1275 | .000 | .941 | .060 |
| 1 | Free to vary | Ethnicity | 2838.26 | 530 | .000 | .936 | .066 |
| 2 | Equal |  | 2479.87 | 600 | .000 | .948 | .056 |
| T3 |  |  |  |  |  |  |  |
| 1 | Free to vary | Gender | 2244.52 | 530 | .000 | .926 | .066 |
| 2 | Equal |  | 2060.66 | 600 | .000 | .937 | .057 |
| 1 | Free to vary | Age | 2918.37 | 1060 | .000 | .930 | .069 |
| 2 | Equal |  | 2944.26 | 1275 | .000 | .938 | .060 |
| 1 | Free to vary | Ethnicity | 2078.26 | 530 | .000 | .940 | .062 |
| 2 | Equal |  | 1898.12 | 600 | .000 | .950 | .054 |
